# Use of machine learning models to predict prognosis of combined pulmonary fibrosis and emphysema in a Chinese population

**DOI:** 10.1186/s12890-022-02124-6

**Published:** 2022-08-29

**Authors:** Qing Liu, Di Sun, Yu Wang, Pengfei Li, Tianci Jiang, Lingling Dai, Mengjie Duo, Ruhao Wu, Zhe Cheng

**Affiliations:** Department of Respiratory and Critical Care Medicine, The First Affiliated Hospital of Zhengzhou University, Zhengzhou, People’s Republic of China

**Keywords:** CPFE, Machine learning model, Nomogram, ILD-GAP model, Prognosis

## Abstract

**Background:**

Combined pulmonary fibrosis and emphysema (CPFE) is a novel clinical entity with a poor prognosis. This study aimed to develop a clinical nomogram model to predict the 1-, 2- and 3-year mortality of patients with CPFE by using the machine learning approach, and to validate the predictive ability of the interstitial lung disease-gender-age-lung physiology (ILD-GAP) model in CPFE.

**Methods:**

The data of CPFE patients from January 2015 to October 2021 who met the inclusion criteria were retrospectively collected. We utilized LASSO regression and multivariable Cox regression analysis to identify the variables associated with the prognosis of CPFE and generate a nomogram. The Harrell's C index, the calibration curve and the area under the receiver operating characteristic (ROC) curve (AUC) were used to evaluate the performance of the nomogram. Then, we performed likelihood ratio test, net reclassification improvement (NRI), integrated discrimination improvement (IDI) and decision curve analysis (DCA) to compare the performance of the nomogram with that of the ILD-GAP model.

**Results:**

A total of 184 patients with CPFE were enrolled. During the follow-up, 90 patients died. After screening out, diffusing lung capacity for carbon monoxide (DLCO), right ventricular diameter (RVD), C-reactive protein (CRP), and globulin were found to be associated with the prognosis of CPFE. The nomogram was then developed by incorporating the above five variables, and it showed a good performance, with a Harrell's C index of 0.757 and an AUC of 0.800 (95% CI 0.736–0.863). Moreover, the calibration plot of the nomogram showed good concordance between the prediction probabilities and the actual observations. The nomogram also improved the discrimination ability of the ILD-GAP model compared to that of the ILD-GAP model alone, and this was substantiated by the likelihood ratio test, NRI and IDI. The significant clinical utility of the nomogram was demonstrated by DCA.

**Conclusion:**

Age, DLCO, RVD, CRP and globulin were identified as being significantly associated with the prognosis of CPFE in our cohort. The nomogram incorporating the 5 variables showed good performance in predicting the mortality of CPFE. In addition, although the nomogram was superior to the ILD-GAP model in the present cohort, further validation is needed to determine the clinical utility of the nomogram.

## Introduction

Pulmonary interstitial fibrosis and emphysema are two distinct clinical entities with different pathogeneses and pathophysiologic manifestations. However, an increasing number of studies consider that the two phenotypes can coexist within one patient [1, 2]. Cottin et al. defined a novel phenotype, “combined pulmonary fibrosis and emphysema (CPFE)”, in 2005 [3]. CPFE is a clinical syndrome characterized by the coexistence of emphysema in the upper zones and fibrosis in the bases of the lungs [1, 2]. The median survival time for CPFE patients is reported to be 2.1 to 6.1 years, which is extremely poorer than that of patients with fibrosis or emphysema alone [4, 5]. Therefore, a validated risk assessment is desperately needed for the cognition and management of CPFE patients.

The study of prognostic prediction of CPFE remains challenging because of the heterogeneity in disease-specific variables and the lack of awareness for this clinical entity [5–9]. Unfortunately, research evaluating and establishing a prognostic prediction system for CPFE is rare to date. The interstitial lung disease-gender-age-lung physiology (ILD-GAP) model is widely used to predict the prognosis of chronic ILD subtypes, including idiopathic pulmonary fibrosis (IPF), connective tissue disease associated ILD (CTD-ILD) and unclassifiable ILD [10]. The previous researches indicated that the prevalence of emphysema was around 27% in patients with chronic ILD subtypes, including IPF and CTD-ILD [11]. CPFE is a distinct chronic ILD subtype with special clinical features and a poor prognosis [3]. However, previous studies have not performed CPFE subtyping analysis with the ILD-GAP model. Therefore, there is an urgent need to explore the prognostic factors of CPFE and to assess and improve the ILD-GAP model [11].

In this study, we investigated the prognostic factors of CPFE and established a comprehensive nomogram to predict the mortality of CPFE in a Chinese population. Furthermore, we also evaluated the prognostic predictive performance of the ILD-GAP model in CPFE.

## Methods

### Study population

This study was a retrospective cohort study that included 184 confirmed CPFE patients who were admitted to the First Affiliated Hospital of Zhengzhou University between January 2015 and October 2021.

Patients were diagnosed with CPFE according to the criteria suggested by Cottin et al. [3], namely, the radiographic presence of centrilobular and/or paraseptal emphysema (≥ 10%) in the upper zones and pulmonary fibrosis in the bases of the lungs.

CTD was defined according to the criteria recommended by the American Rheumatism Association and the American College of Rheumatology [12–19], including systemic sclerosis (SSc), rheumatoid arthritis (RA), polymyositis/dermatomyositis (PM/DM), sjogren syndrome (SS), ankylosing spondylitis (AS), systemic lupus erythematosus (SLE), antineutrophil cytoplasmic antibody (ANCA)-associated vasculitis (AAV), mixed connective tissue disease (MCTD), and undifferentiated connective tissue disease (UCTD).

The exclusion criteria were as follows: (1) patients who met the criteria for the diagnosis of CPFE, but CPFE was secondary to other etiologies, including pneumoconiosis (asbestosis or siderosis); (2) patients with incomplete data; and (3) patients younger than 18 years old.

### Ethics issue

The ethical approval of this study was granted by the Ethics Committee of Scientific Research and Clinical Trials of the First Affiliated Hospital of Zhengzhou University (approval number: 2019-KY-116) prior to the data collection. Since the data were deidentified and aggregated, written consent was waived.

### Data collection

Data were collected from electronic medical records at the initial diagnosis. The collected data included demographic characteristics, systematic classification, pulmonary function test results, echocardiography results, high-resolution computed tomography (HRCT) images and laboratory test results.

The demographic characteristics included age, sex, body mass index, smoking history, complications (lung cancer and pulmonary hypertension) and treatment. Pulmonary hypertension (PH) was defined according to the echocardiographic criteria for high probability of PH recommended by the European Society of Cardiology and the European Respiratory Society (ESC/ERS): the peak tricuspid regurgitation velocity (TRV) > 3.4 m/s; or the TRV is 2.9–3.4 m/s within the signs assessing the right ventricular (RV) size, the pressure overload, the pattern of blood flow velocity out of the RV, the diameter of the pulmonary artery and an estimate of right atrial pressure [20].

The systematic classifications included idiopathic CPFE and CTD-CPFE, using the classification criteria of CTD as the inclusion standard.

The variables of the pulmonary function test that we collected included the percentage of the predicted values (%Predicted) for forced expiratory volume in the first second (FEV_1_), forced vital capacity (FVC), total lung capacity (TLC), peak expiratory flow (PEF), maximal midexpiratory flow rate (MMEF, also known as FEF 25–75), DLCO and the ratio of FEV_1_ to FVC (FEV_1_/FVC).

The collected echocardiography data included right atrial area (RAA), RVD (from the right ventricular four-chamber view, the straight line joining the midpoint of the tricuspid valve annulus to the right ventricular apex in end-diastole constituted the RVD) [21, 22], left atrial area, left ventricular end diastolic diameter, ascending aortic diameter, aortic annulus diameter, pulmonary artery diameter, pulmonary regurgitant peak velocity, and left ventricular ejection fraction (LVEF).

HRCT scans were examined by two independent chest radiologists, and final conclusions on the findings were reached by consensus. The collected HRCT images included fine reticular opacity, ground-glass opacity, pseudoplaque, flocculent shadow (any area that preferentially attenuates the X-ray beam and therefore appears more opaque than the surrounding area), parenchymal band, honeycomb shadow, traction bronchiectasis, local pleural thickening and mediastinal lymphadenopathy. The detailed description of these images refers to the Fleischner terminology [23].

The collected laboratory examination data included leukocyte count, erythrocyte count, haemoglobin count, platelet count, red cell distribution width, platelet distribution width, aspartate transaminase, alanine aminotransferase, γ glutamyl transferase, alkaline phosphatase, total protein, albumin (ALB), globulin, total bilirubin, direct bilirubin, indirect bilirubin, urea nitrogen, creatinine, uric acid, glomerular filtration rate, total cholesterol, total triglycerides, high-density lipoprotein, low-density lipoprotein, B-type natriuretic peptide, CRP, procalcitonin, erythrocyte sedimentation rate, complement component C3, complement component C4, immunoglobulin A, immunoglobulin M, immunoglobulin G, Krebs von den Lungen-6 (KL-6), partial pressure of carbon dioxide, partial pressure of arterial oxygen, blood oxygen saturation, lactate, alpha-fetoprotein, carcinoembryonic antigen, carbohydrate antigen 125, cytokeratin-19-fragment (CYFRA21-1), neuron-specific enolase, carbohydrate antigen 199, carbohydrate antigen 153, carbohydrate antigen 724 and serum ferritin.

### Follow-up and outcome assessment

The study endpoint was all-cause mortality during follow-up until January 2022. Follow-up information was obtained from patients or their families via telephone interviews.

### Statistical analysis

Missing data were processed by multiple imputations. Imputation for missing variables was considered if missing values were less than 20%. A t test or corrected t test was used to compare the continuous variables of normal distribution between the two groups, which were presented as the mean ± standard deviation (mean ± SD). Continuous nonnormally distributed data were compared using the Mann–Whitney U test and presented as the median and interquartile range (IQR, 25–75th percentiles). Categorical variables of the two groups were compared by the χ2 test and presented as frequencies (percentages). LASSO regression analysis was used for data dimension reduction and variable selection. The penalty value (λ value) was selected by tenfold cross-validation, and the best subset of the variables was selected by using the “glmnet” package of R. The significance of each variable in the best subset was evaluated by univariable Cox regression analysis. The variables with *P* values less than 0.05 were entered into the forward stepwise regression multivariable Cox analysis. A nomogram was constructed based on the results of multivariate Cox regression analysis and by using the “rms” package of R. For clinical use of the model, the risk scores of each patient were calculated based on the nomogram. The performance of the nomogram was assessed by discrimination and calibration [24]. The discriminative ability of the model was determined by the area under the receiver operating characteristic (ROC) curve (AUC). In addition, the nomogram was subjected to 1000 bootstrap resamples for internal validation to assess its predictive accuracy [25]. The calibration of the internal validation model was performed by a visual calibration plot comparing the predicted and actual probability of mortality. The ILD-GAP stage was calculated based on gender (0–1 points), age (0–2 points), and two physiologic lung function parameters—FVC and DLCO (0–5 points) [10]. The predictive performance of the nomogram and ILD-GAP model were evaluated by a likelihood ratio test (using “lmtest” R package), NRI and IDI (using “survC1” and “survIDINRI” R package), the comparison of the Harrell's C index (using “survival” R package) and AUC values (using “ROCR” R package). Finally, decision curve analysis (DCA) was performed by the source file “stdca. R”. All analyses were performed using SPSS version 26.0 and R version 4.1.1. For all the analyses, *P* < 0.05 was considered to be statistically significant.

## Results

### Clinical characteristics

**Fig. 1:**
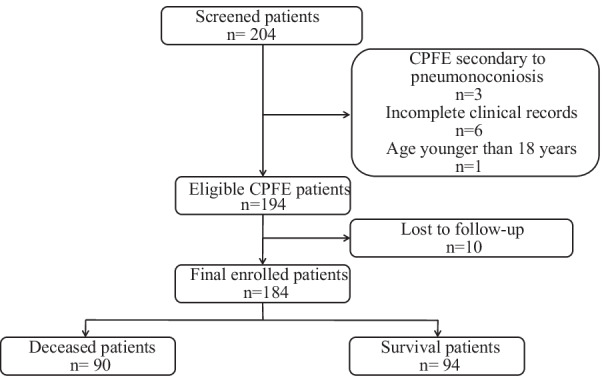
Flowchart of the patients included in the analysis. Abbreviations: CPFE: combined pulmonary fibrosis and emphysema

A total of 204 patients with confirmed CPFE were screened in the present study according to the above defined criteria. After excluding the patients with CPFE secondary to pneumonoconiosis (n = 3), patients with incomplete data (n = 6), those younger than 18 years old (n = 1) and those lost to follow-up (n = 10), 184 patients were included in this study, as presented in Fig. 1. During follow-up (median duration 16.9 months), a total of 90 (48.9%) patients died. The clinical characteristics of all patients in the study are shown in Table 1 (at the end of the article). In our study cohort, 143 (78%) were male, 105 (57%) had a history of smoking, and the mean age at the initial diagnosis was 67 ± 11 years old. The median overall survival time was 32.8 months. Compared with the surviving patients, the deceased patients were significantly older, were more likely to have pulmonary hypertension and lung cancer, and treated without acetylcysteine (all *P* < 0.05). Compared with patients who were alive, those who died were more likely to have lower FVC, TLC, DLCO, LVEF and higher RVD (all *P* < 0.05). Additionally, the deceased patients were more likely to have mediastinal lymphadenopathy, higher levels of serum KL-6 and CYFRA21-1 (all *P* < 0.05).

**Table 1:** Clinical characteristics of the CPFE patients

Variable	All patients (n = 184)	Deceased patients (n = 90)	Surviving patients (n = 94)	*P* value*
Age, y	66.8 ± 10.5	69.4 ± 9.2	64.2 ± 11.0	0.001
*Sex, n (%)*				
Male	143 (77.7)	71 (78.9)	72 (76.6)	0.709
Female	41 (22.3)	19 (21.1)	22 (23.4)	
BMI, kg/m^2^	23.6 (21.5, 25.6)	23.4 (21.4, 25.0)	23.7 (21.5, 26.0)	0.379
Smoking, n (%)	105 (57.1)	51 (56.7)	54 (57.5)	0.915
*Comorbidities, n (%)*				
Pulmonary hypertension	48 (26.1)	33 (36.7)	15 (16.0)	0.001
Lung cancer	6 (3.3)	6 (6.7)	0 (0.0)	0.033
*Baseline lung function*				
FEV_1_, %Predicted	82.8 (72.4, 90.0)	82.3 (71.5, 87.9)	84.0 (73.5, 96.1)	0.099
FVC, %Predicted	84.0 (72.4, 91.1)	82.9 (66.0, 89.1)	86.1 (74.8, 96.4)	0.025
FEV_1_/FVC, %	77.9 (71.0, 85.3)	78.0 (72.8, 83.7)	77.9 (68.5, 85.7)	0.574
TLC, %Predicted	74.2 (66.4, 80.3)	73.1 (63.8, 78.4)	74.6 (68.5, 83.0)	0.034
DLCO, %Predicted	44.0 (36.0, 52.1)	40.3 (27.0, 47.2)	46.7 (40.7, 58.6)	< 0.001
*Echocardiography*				
RAA, cm^2^	14.0 ± 3.6	14.5 ± 4.5	13.7 ± 2.4	0.135
RVD, mm	17.3 ± 3.3	18.5 ± 4.0	16.1 ± 1.8	< 0.001
LVEF, %	63.0 (61.0, 64.0)	62.0 (60.0, 64.0)	63.1 (61.8, 65.0)	0.027
*Image of HRCT, n (%)*				
Fine reticular opacity	94 (51.1)	50 (55.6)	44 (46.8)	0.235
Ground-glass opacity	15 (8.2)	7 (7.8)	8 (8.5)	0.856
Patchy shadow	89 (48.4)	47 (52.2)	42 (44.7)	0.306
Traction bronchiectasis	16 (8.7)	6 (6.7)	10 (10.6)	0.339
Honeycomb shadow	43 (23.4)	23 (25.6)	20 (21.3)	0.493
Local pleural thickening	146 (79.3)	76 (84.4)	70 (74.5)	0.095
Mediastinal lymphadenopathy	115 (62.5)	64 (71.1)	51 (54.3)	0.018
KL-6, U/ml	1201.6 (830.0, 1263.8)	1217.9 (1095.8, 1296.4)	1175.5 (696.5, 1262.0)	0.028
CYFRA21-1, ng/ml	3.8 (2.3, 5.7)	4.2 (2.7, 6.4)	3.0 (2.1, 5.3)	0.015
*Treatment, n (%)*				
Pirfenidone	76 (41.3)	36 (40.0)	40 (42.6)	0.725
Glucocorticoids	86 (46.7)	46 (51.1)	40 (42.6)	0.245
Acetylcysteine	118 (64.1)	51 (56.7)	67 (71.3)	0.039
Immunosuppressive agents	27 (14.7)	15 (16.7)	12 (12.8)	0.455
Follow-up time, months	16.9 (6.7, 33.3)	9.4 (2.3, 21.7)	21.0 (13.2, 38.6)	< 0.001

*CPFE* combined pulmonary fibrosis and emphysema; *mean* ± *SD* mean ± standard deviation; *BMI* body mass index; *IQR* interquartile range; *FEV1* forced expiratory volume in the first second; *FVC* forced vital capacity; *FEV1/FVC* the ratio of FEV1 to FVC; *TLC* total lung capacity; *DLCO* diffusing lung capacity for carbon monoxide; *RAA* right atrial area; *RVD* right ventricular diameter; *LVEF* left ventricular ejection fraction; *HRCT* high-resolution computed tomography; *KL-6* Krebs von den Lungen-6; *CYFRA21-1* cytokeratin-19-fragment

*Comparison of the performance of the deceased patients and the surviving patients for clinical characteristics

Data were presented as the means ± SDs, numbers (%) or medians (IQR)

### Model establishment

**Fig. 2:**
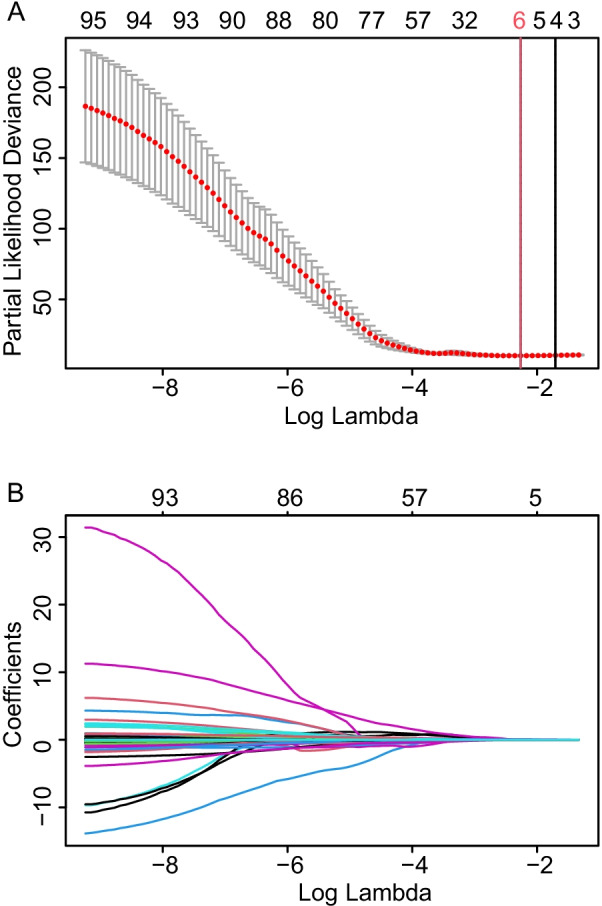
In the least absolute shrinkage and selection operator (LASSO) model, the minimum standard was adopted to obtain the value of the super parameter λ by tenfold cross-validation. The λ value was confirmed as 0.104 (log(lambda): − 2.262), where the optimal lambda resulted in 6 nonzero coefficients. **A** Six risk factors selected using LASSO regression analysis. Solid vertical lines were drawn at the optimal values using the minimum criteria (red line) and the 1 standard error of the minimum criteria (black line) (at minimum criteria including Age, DLCO, RVD, CRP, Albumin and Globulin). **B** LASSO coefficient profiles of the 95 risk factors. Abbreviations: RVD, right ventricular diameter; DLCO, diffusing lung capacity for carbon monoxide; CRP, C-reactive protein

**Fig. 3:**
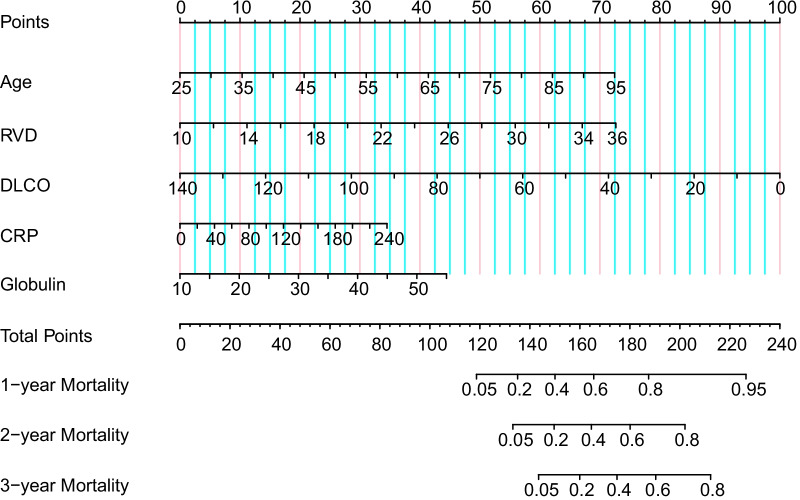
Nomogram for predicting the 1-, 2- and 3-year mortality of CPFE. The points of each feature were added to obtain the total points, and the corresponding 1-, 2- and 3-year mortality was obtained based on the total points. Abbreviations: CPFE, combined pulmonary fibrosis and emphysema; RVD, right ventricular diameter; DLCO, diffusing lung capacity for carbon monoxide; CRP, C-reactive protein

Ninety-five prognostic variables were enrolled in this study. First, we reduced the dimension and selected the best prognostic subset of these indicators by LASSO regression analysis. Then, a ten-fold cross validation of the LASSO model was performed for tuning parameter selection via the minimum criteria (Fig. 2A). The track of each prognostic indicator coefficient was observed in the LASSO coefficient profiles with the changing of the log (lambda) in the LASSO algorithm (Fig. 2B). The optimal lambda value was 0.104 (log(lambda): − 2.262) using the LASSO algorithm, and 6 variables were selected as potential influencing factors of prognosis—age, DLCO, RVD, CRP, ALB and globulin. To explore the potential influencing factors associated with the prognosis of CPFE, we further conducted univariate and multivariate Cox regression analyses. Univariable Cox regression analysis revealed that increased age (HR 1.053, 95% CI 1.031–1.076), RVD (HR 1.115, 95% CI 1.074–1.158), CRP (HR 1.009, 95% CI 1.005–1.013) and globulin (HR 1.066, 95% CI 1.038–1.094) were correlated with a higher mortality risk (all *P* < 0.001) (Table 2). However, higher DLCO (HR 0.965, 95% CI 0.952–0.978) and ALB (HR 0.906, 95% CI 0.867–0.948) were correlated with a lower mortality risk (both *P* < 0.001) (Table 2). Significant indicators (*P* value < 0.05) in the univariate analysis were entered into a multivariate Cox model, and the results showed that the 5 variables of age, DLCO, RVD, CRP and globulin affected all-cause mortality significantly (all *P* < 0.05) (Table 2). The nomogram for prognostic prediction was established according to the results of the multivariable Cox regression analysis (Fig. 3).

**Table 2:** Univariate and multivariate Cox analyses for overall mortality in CPFE

Variables	Univariable COX regression analysis		Multivariable COX regression analysis	
	Hazard ratio (95% CI)	*P* value	Hazard ratio (95% CI)	*P* value
Age, years	1.053 (1.031–1.076)	< 0.001	1.040 (1.017–1.063)	< 0.001
DLCO, %Predicted	0.965 (0.952–0.978)	< 0.001	0.973 (0.958–0.989)	0.001
RVD, mm	1.115 (1.074–1.158)	< 0.001	1.111 (1.064–1.161)	< 0.001
CRP, mg/L	1.009 (1.005–1.013)	< 0.001	1.005 (1.001–1.010)	0.023
Globulin, g/L	1.066 (1.038–1.094)	< 0.001	1.038 (1.005–1.072)	0.023
ALB, g/L	0.906 (0.867–0.948)	< 0.001		
ILD-GAP model	1.652 (1.391–1.962)	< 0.001		

*CPFE* combined pulmonary fibrosis and emphysema; *DLCO* diffusing lung capacity for carbon monoxide; *RVD* right ventricular diameter; *CRP* C-reactive protein; *ALB* albumin

### Performance of the model

**Fig. 4:**
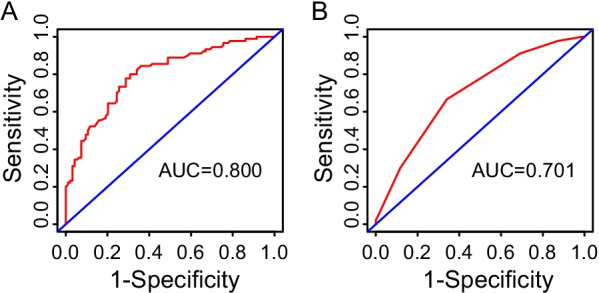
The ROC curves with AUCs of 0.800 and 0.701 to demonstrate the discriminatory ability of the two models. **A** The ROC curve with an AUC of 0.800 of the nomogram. The red line represents the discriminatory ability of the nomogram; the blue line represents the reference line. **B** The ROC curve with an AUC of 0.701 of the ILD-GAP model. The red line represents the discriminatory ability of the ILD-GAP model; the blue line represents the reference line

**Fig. 5:**
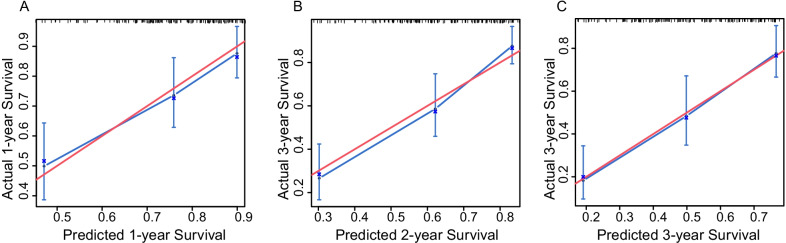
Calibration plot of the nomogram showing predicted 1-year (**A**), 2-year (**B**) and 3-year (**C**) survival by stage against actual survival

The predictive performance of the nomogram was good in our study cohort. The C-index value was 0.757, and the mean Harrell's C index in the validation cohort constructed by 1000 bootstrap resamples was 0.853. The AUC of the nomogram was 0.800 (95% CI 0.736–0.863) (Fig. 4A). The calibration curves of the nomogram showed high consistency between the predicted and the actual 1-, 2- and 3-year survival probabilities in our study cohort (Fig. 5).

**Fig. 6:**
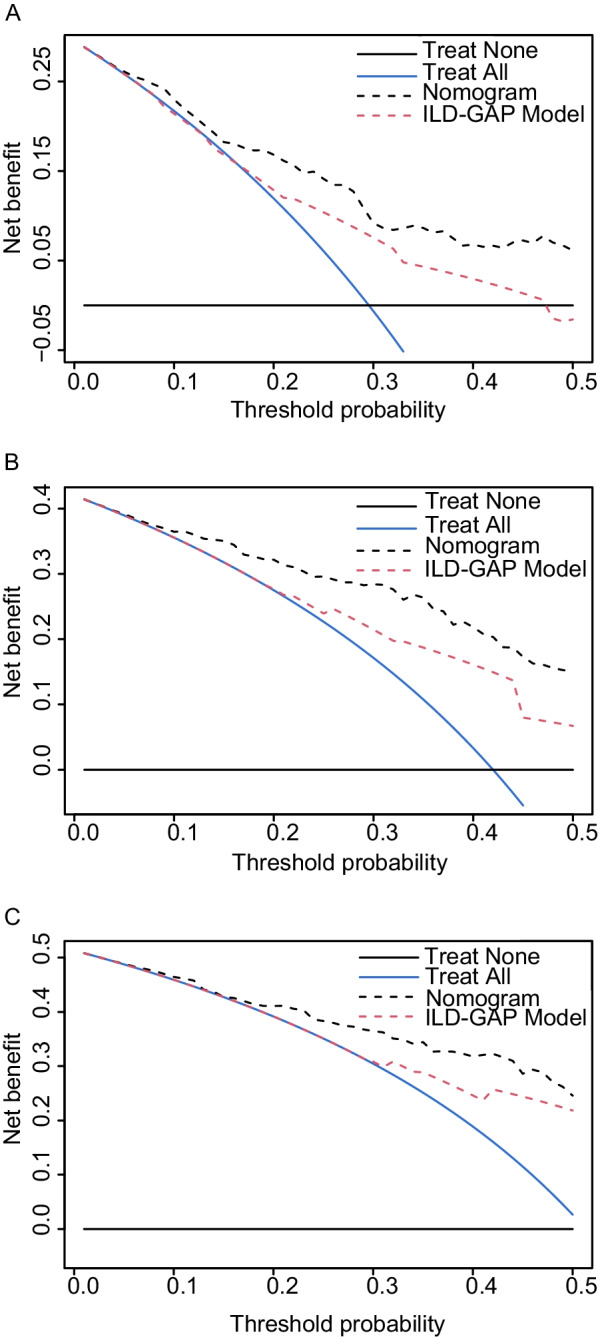
Decision curve analysis comparing the clinical performance of the nomogram and ILD-GAP model. For the risk of 1-year (**A**), 2-year (**B**) and 3-year (**C**) mortality, the nomogram showed the highest net benefit for all potential thresholds. The black dotted line represents the nomogram, and the red dotted line represents the ILD-GAP model. The blue line represents the assumption that all patients have been treated, and the black line represents the assumption that no patients have been treated

The ILD-GAP model exhibited increasing mortality risk in patents with higher scores by univariate variable Cox regression (HR 1.652, 95% CI 1.391–1.962, *P* < 0.001; Table 2). The C-index value was 0.657 of the ILD-GAP model, which was lower than that of the nomogram (0.757). The likelihood ratio test showed that there was a statistically significant enhancement of the predictive performance when the inclusion of nomogram in the ILD-GAP model (*P* < 0.001), but there was no significant difference when the inclusion of the ILD-GAP model in nomogram (*P* = 0.160) (Table 3). Moreover, the NRIs of the nomogram and the ILD-GAP model for 1-, 2- and 3-year mortality were 0.332 (95% CI 0.086–0.476, *P* = 0.013), 0.362 (95% CI 0.087–0.511, *P* = 0.020) and 0.173 (95% CI − 0.069 to 0.381, *P* = 0.120), respectively, and the IDIs of the nomogram and the ILD-GAP model for 1-, 2- and 3-year mortality were 0.145 (95% CI 0.054–0.213, *P* < 0.001), 0.142 (95% CI 0.057–0.230, *P* < 0.001) and 0.084 (95% CI − 0.030 to 0.175, *P* = 0.133), respectively (Table 4). These results indicated that the nomogram showed a better prognostic performance than the ILD-GAP model in the present cohort. Then, we performed DCA to evaluate the net clinical benefit that the nomogram would bring to patients compared with the ILD-GAP model. In this study, the nomogram showed a better net benefit than the ILD-GAP model for clinical intervention for the optimal decision threshold > 0% (Fig. 6).

**Table 3:** Likelihood ratio test between the nomogram and the ILD-GAP model

	Nomogram	ILD-GAP model	Nomogram + ILD-GAP model
Likelihood ratio	78.57	33.51	80.55
*P* value	0.160*	< 0.001#	

*Comparison of the performance of mortality by nomogram only with that of the combination of the nomogram and the ILD-GAP model

^#^Comparison of the performance of mortality by the LID-GAP model only with that of the combination of the nomogram and the ILD-GAP model

**Table 4:** NRI and IDI of the nomogram and the ILD-GAP model in mortality prediction

	NRI (95% CI)	*P* value	IDI (95% CI)	*P* value
1-Year mortality	0.332 (0.086–0.476)	0.013	0.145 (0.054–0.213)	< 0.001
2-Year mortality	0.362 (0.087–0.511)	0.020	0.142 (0.057–0.230)	< 0.001
3-Year mortality	0.173 (− 0.069 to 0.381)	0.120	0.084 (− 0.030 to 0.175)	0.133

*NRI* net reclassification improvement; *IDI* integrated discrimination improvement

## Discussion

CPFE is a clinical syndrome without full recognition that is characterized by progressive worsening respiratory symptoms and markedly impaired lung diffusion function [2, 3, 26]. Unfortunately, limited studies have reported its prognostic risk factors [7, 27]. Moreover, none of the previous studies developed a prognostic predictive system for CPFE patients. In the present study, we explored the clinical characteristics and prognostic features of CPFE patients based on the real-world data. Then, we incorporated 5 optimal prognostic variables into a user-friendly nomogram for predicting the prognosis of CPFE. We also performed a series of validations to evaluate the predictive performance of the ILD-GAP model.

We used LASSO regression to screen out 6 variables from the 95 candidates by examining the predictor-outcome association by shrinking the regression coefficients. LASSO is a method for dimension reduction and variable selection, and the number of selected predictors is not limited by the sample size when the number of samples is more than the number of the variables [28, 29]. There were 95 variables in 180 samples of this study; therefore, LASSO regression was a proper and credible method to establish the model. Then, we used multivariate Cox regression analysis to substantiate the prognostic value of the 6 variables. The results of multivariable Cox analysis showed that age, DLCO, RVD, and the levels of serum CRP and globulin were significantly associated with the prognosis of CPFE.

Age has been reported as a risk factor for the prognosis of CPFE and many other lung diseases because older individuals typically have more comorbidities and poorer health status [30]. CPFE has a mixed pattern of pulmonary function that preserved lung volumes associated with disproportionately reduced DLCO [3]. The cause may be the reduction of normally functioning alveolar capillary units and pulmonary capillary blood volume, which reduces the effective surface area available for gas exchange [2]. In addition, alveolar membrane thickening, excessive accumulation of extracellular matrix and alveolar epithelial cell damage may also be involved in the process of decreasing DLCO according to past studies [2, 31]. Consistently, in this study, a lower DLCO was demonstrated to be associated with the prognosis of CPFE. We also found that RVD was associated with poor prognosis in our study. Ventricular remodelling, mainly showing ventricular wall hypertrophy and cardiac dilatation, is one of the mechanisms of heart failure. Moreover, most patients complicated with heart failure show a reduced cardiac index, which is the most accurate prognostic determinant of CPFE [32]. Previous studies showed that pulmonary hypertension (PH) had a higher prevalence in patients with CPFE with a poor prognosis [33]. In our study, PH was also associated with worse outcomes (HR 2.093, 95% CI 1.360–3.222, *P* < 0.05) in the univariable Cox regression analysis. However, PH was not selected in the LASSO regression analysis. The probable reason is that RVD, which is essentially a marker of right ventricular dilation, may be collinear with PH given that right ventricular dilation is often a consequence of PH. With the progression of PH, the pulmonary artery systolic pressure will decrease with the occurrence of right heart failure, while right ventricular dilation is irreversible.

CRP and globulin are the serological indicators commonly tested in clinic, and their levels are acceptably correlated with the severity of infection and immune status [34]. Our study demonstrated that serum CRP and globulin were significantly associated with increased CPFE mortality. CRP is synthesized in the liver as a result of several stimuli, e.g., interleukin (IL)-6, and is also considered to be a classic acute-phase protein [35, 36]. Several studies have identified that airway damage and acute exacerbations of infection are the main reasons for the poor prognosis for CPFE [2, 27], which could lead to an increase in CRP, consistent with our results. In addition, CPFE could be involved in multiple systems and organs by self-directed inflammation, commonly leading to collagen deposition and tissue damage [37, 38]. CRP is a pattern-recognition molecule of the innate immune system, and its binding to ligands can mediate direct interactions with immunoglobulin receptors and trigger classic complement activation, which is related to the pathogenesis and progression of CPFE [39, 40]. Immune dysregulation is a driver of both idiopathic CPFE and CTD-CPFE [38, 41], and the increasing concentration of immunoglobulin is acceptably related to the active phase and poor prognosis of CPFE [40]. The serum level of globulin could reflect the increase of immunoglobulin and some other abnormal circulating antibodies to some extent.

A nomogram can provide an individualized, evidence-based and highly accurate risk estimation, thus facilitating decision-making by physicians and policy makers [42, 43]. The nomogram we constructed demonstrated good discrimination as assessed by Harrell's C index (0.757) and AUC value (0.800). The optimal calibration curves indicate good consistency between the predicted probabilities and the actual observations, although the variance around the three points shown is high in Fig. 5, which may be due to the relatively small sample size. However, to our knowledge, our sample size was the largest for the study of the prognosis of CPFE, and the visual representation of the relationship between predicted and observed prognoses was the best way to evaluate calibration [44]. The ILD-GAP model had a good predictive ability in chronic ILD subtypes, but the coexistence of CPFE in IPF/CTD-ILD may affect the existing assessment model [45]. In our cohort, the Harrell's C index of the ILD-GAP model was only 0.657, which indicated poorer discriminative ability than the nomogram (0.757). Moreover, although the combination of the nomogram and the ILD-GAP model was superior to the ILD-GAP model alone, it was not superior to the nomogram alone. The nomogram also improved the discrimination ability compared to the ILD-GAP model substantiated by the NRI and IDI. DCA has been widely employed to substantiate the clinical utility and benefit when the predictive model guides clinical practice [46]. The DCA proved that when the decision threshold was > 0%, using the nomogram in the current study showed a higher net benefit than using the ILD-GAP model for clinical intervention. We believe this is because of comorbid emphysema, which may impact survival independent of ILD severity [10]. Previous research indicated that FEV_1_ and FEV_1_/FVC were mortality predictors of pulmonary emphysema [47], while there were no significant differences between the deceased and surviving groups. The possible reason may be that we collected the data at the initial diagnosis of CPFE. At the initial stage of the disease, the hyperinflation and high compliance of emphysema probably compensate for the volume loss, which presents relatively preserved lung volume, while even mild emphysema may have an additive effect with fibrosis on the progression of the disease and affect the prognosis of CPFE [3]. The other reason why the nomogram in the current study showed a higher net benefit than the ILD-GAP model may be that there were prognostically important variables for this population that were not captured in the ILD-GAP model. The variables in the nomogram are comprehensive and acquired easily, and can be widely applied to clinical practice after further validation and improvement. Although the predictive ability of the ILD-GAP model in CPFE was not superior, the model has been widely adopted to predict the mortality of chronic ILD due to its conciseness and established performance [10].

Although our study is based on real-world data and has relatively complete information of the patients, there are still some limitations. First, this retrospective cross-sectional analysis was based on data from a single institution and therefore may suffer from selection bias. Therefore, more prospective and longitudinal studies are required to further validate the reliability of the nomogram. Second, the nomogram lacked specific genetic markers. However, our study screened 95 clinical characteristics and then selected the most significant variables associated with the prognosis of CPFE. These variables are comprehensive and easily available, thus facilitating decision-making by physicians. Third, quantitative indicators of fibrosis or emphysema were not included in the study, but the more objective indicators, lung function parameters, were included because of the risk of collinearity [48, 49]. Fourth, the diagnosis of PH in the study was based on echocardiography instead of right heart catheterization (RHC). However, a previous study indicated that echocardiography had a specificity of 100% to identify patients with PH and a negative predictive value of 84.72% to rule out PH [50]. Fifth, owing to their small sample sizes, other subtypes of CPFE, such as pneumoconiosis-associated CPFE, were not enrolled to maintain the consistency of baseline data as much as possible. To the best of our knowledge, this is the first model established for predicting the overall mortality of CPFE based on a large sample, and we believe that an early report is urgent and crucial to provide a basis for further studies.

## Conclusion

In this study, age, DLCO, RVD, CRP, and globulin were identified as significant predictive factors of prognosis for CPFE patients. Then, we established a nomogram incorporating the 5 variables to predict the mortality of CPFE, and the nomogram showed good performance. In addition, the nomogram was superior to the ILD-GAP model in terms of performance in the present cohort. Finally, although our nomogram can facilitate individualized therapy design, further validation is still needed to determine the clinical utility of the nomogram.

## Data Availability

All data generated or analyzed during this study are included in this published article. Besides, any additional data/files may be obtained from the corresponding author on reasonable request.

## Abbreviations

CPFE: Combined pulmonary fibrosis and emphysema
ILD-GAP: Interstitial lung disease-gender-age-lung physiology
AUC: Area under the curve
NRI: Net reclassification improvement
IDI: Integrated discrimination improvement
DCA: Decision curve analysis
DLCO: Diffusing lung capacity for carbon monoxide
RVD: Right ventricular diameter
CRP: C-reactive protein
IPF: Idiopathic pulmonary fibrosis
CTD-ILD: Connective tissue disease associated ILD
CTD-CPFE: CTD-ILD associated CPFE
SSc: Systemic sclerosis
RA: Rheumatoid arthritis
PM/DM: Polymyositis/Dermatomyositis
SS: Sjogren syndrome
AS: Ankylosing spondylitis
SLE: Systemic lupus erythematosus
ANCA-AAV: Antineutrophil cytoplasmic antibody-associated vasculitis
MCTD: Mixed connective tissue disease
UCTD: Undifferentiated connective tissue disease
HRCT: High-resolution computed tomography
%Predicted: The percentage predicted values
FEV_1_: Forced expiratory volume in the first second
FVC: Forced vital capacity
TLC: Total lung capacity
PEF: Peak expiratory flow
MMEF: Maximal midexpiratory flow rate
FEV_1_/FVC: The ratio of FEV_1_ to FVC
RAA: Right atrial area
LVEF: Left ventricular ejection fraction
ALB: Albumin
KL-6: Klebs von den Lungen-6
CYFRA21-1: Cytokeratin-19-fragment
Mean ± SD: Mean ± standard deviation
IQR: Interquartile range (25–75th percentiles)
